# Evaluation of Web-Based and In-Person Methods to Recruit Adults With Type 1 Diabetes for a Mobile Exercise Intervention: Prospective Observational Study

**DOI:** 10.2196/28309

**Published:** 2021-07-08

**Authors:** Garrett I Ash, Stephanie Griggs, Laura M Nally, Matthew Stults-Kolehmainen, Sangchoon Jeon, Cynthia Brandt, Barbara I Gulanski, Elias K Spanakis, Julien S Baker, Robin Whittemore, Stuart A Weinzimer, Lisa M Fucito

**Affiliations:** 1 Pain, Research, Informatics, Medical Comorbidities and Education Center Veterans Affairs Connecticut Healthcare System West Haven, CT United States; 2 Center for Medical Informatics Yale University School of Medicine New Haven, CT United States; 3 Frances Payne Bolton School of Nursing Case Western Reserve University Cleveland, OH United States; 4 Section of Pediatric Endocrinology & Diabetes Yale University School of Medicine New Haven, CT United States; 5 Digestive Health Multispecialty Clinic Yale-New Haven Hospital New Haven, CT United States; 6 Department of Biobehavioral Sciences Teachers College Columbia University New York, NY United States; 7 School of Nursing Yale University Orange, CT United States; 8 Section of Endocrinology Veterans Affairs Connecticut Healthcare System West Haven, CT United States; 9 Section of Endocrinology Yale University School of Medicine New Haven, CT United States; 10 Division of Endocrinology Baltimore Veterans Administrative Medical Center Baltimore, MD United States; 11 Division of Endocrinology, Diabetes, and Nutrition University of Maryland School of Medicine Baltimore, MD United States; 12 Department of Sport, Physical Education and Health Hong Kong Baptist University Kowloon Tong China (Hong Kong); 13 Department of Psychiatry Yale University School of Medicine New Haven, CT United States; 14 Yale Cancer Center New Haven, CT United States; 15 Smilow Cancer Hospital Yale-New Haven Hospital New Haven, CT United States

**Keywords:** type 1 diabetes mellitus, exercise, behavior and behavior mechanisms, mobile phone

## Abstract

**Background:**

Our clinical trial of a mobile exercise intervention for adults 18 to 65 years old with type 1 diabetes (T1D) occurred during COVID-19 social distancing restrictions, prompting us to test web-based recruitment methods previously underexplored for this demographic.

**Objective:**

Our objectives for this study were to (1) evaluate the effectiveness and cost of using social media news feed advertisements, a clinic-based approach method, and web-based snowball sampling to reach inadequately active adults with T1D and (2) compare characteristics of enrollees against normative data.

**Methods:**

Participants were recruited between November 2019 and August 2020. In method #1, Facebook and Instagram news feed advertisements ran for five 1-to-8-day windows targeting adults (18 to 64 years old) in the greater New Haven and Hartford, Connecticut, areas with one or more diabetes-related profile interest. If interested, participants completed a webform so that the research team could contact them for eligibility screening. In method #2, patients 18 to 24 years old with T1D were approached in person at clinical visits in November and December 2019. Those who were interested immediately completed eligibility screening. Older patients could not be approached due to clinic restrictions. In method #3, snowball sampling was conducted by physically active individuals with T1D contacting their peers on Facebook and via email for 48 days, with details to contact the research staff to express interest and complete eligibility screening. Other methods referred participants to the study similarly to snowball sampling.

**Results:**

In method #1, advertisements were displayed to 11,738 unique viewers and attracted 274 clickers (2.33%); 20 participants from this group (7.3%) volunteered, of whom 8 (40%) were eligible. Costs averaged US $1.20 per click and US $95.88 per eligible volunteer. Men had lower click rates than women (1.71% vs 3.17%; *P*<.001), but their responsiveness and eligibility rates did not differ. In method #2, we approached 40 patients; 32 of these patients (80%) inquired about the study, of whom 20 (63%) volunteered, and 2 of these volunteers (10%) were eligible. Costs including personnel for in-person approaches averaged US $21.01 per inquirer and US $479.79 per eligible volunteer. In method #3, snowball sampling generated 13 inquirers; 12 of these inquirers (92%) volunteered, of whom 8 (67%) were eligible. Incremental costs to attract inquirers were negligible, and total costs averaged US $20.59 per eligible volunteer. Other methods yielded 7 inquirers; 5 of these inquirers (71%) volunteered, of whom 2 (40%) were eligible. Incremental costs to attract inquirers were negligible, and total costs averaged US $34.94 per eligible volunteer. Demographic overrepresentations emerged in the overall cohort (ie, optimal glycemic control, obesity, and low exercise), among those recruited by news feed advertisements (ie, obesity and older age), and among those recruited by snowball sampling (ie, optimal glycemic control and low exercise).

**Conclusions:**

Web-based advertising and recruitment strategies are a promising means to attract adults with T1D to clinical trials and exercise interventions, with costs comparing favorably to prior trials despite targeting an uncommon condition (ie, T1D) and commitment to an intervention. These strategies should be tailored in future studies to increase access to higher-risk participants.

**Trial Registration:**

ClinicalTrials.gov NCT04204733; https://clinicaltrials.gov/ct2/show/NCT04204733

## Introduction

### Background

Type 1 diabetes (T1D) is characterized by beta cell destruction and absolute deficiency, and it increases the risk of cardiovascular disease among the 1.6 million Americans living with it [1]. There is extensive evidence to endorse exercise as therapy to reduce this risk [2]. Yet, data on optimal strategies to promote exercise safely and successfully among those with T1D who are inadequately active are lacking.

Online programs have potential for improving the scalability, reach, and cost-effectiveness of exercise interventions [3]. Effective behavioral interventions to promote lifestyle change typically involve a skills component, self-monitoring, personalized feedback, and/or an electronic tool and resource to facilitate behavior change [4,5]. While in-person exercise interventions are efficacious for health goals, such as weight loss for people with obesity and no other chronic conditions [6], individuals with T1D must spend several hours per day managing their disease [7], so extra time commitments, such as traveling to exercise, must be minimized.

Quality clinical trials are needed to address the diabetes care needs of adults across the lifespan (18 to 65 years). To have generalizable results, clinical trials must enroll participant samples that represent the target population in terms of sociodemographic and clinical characteristics. Recruiting racially, ethnically, and socioeconomically diverse adults using traditional recruitment strategies is challenging [8]. Another challenge is to recruit a nationally representative sample reflective of adults with T1D to capture those who do not meet glycemic control targets or with other comorbidities and other cardiovascular risk factors such as hypertension. Recruiting through social media has great potential to reach populations who would otherwise not participate in research. Social media is an effective strategy for recruiting young adults—98% use the internet and 88% use social media—and the internet is particularly effective for recruiting young adults aged 18 to 34 years with T1D [9,10]. However, less is known about the effectiveness of social media for recruiting middle-aged to older adults aged 35 to 65 years with T1D.

Social media platforms host numerous T1D support groups that facilitate peer and role model support [11,12], and advertisements through two of these groups—College Diabetes Network and Beyond Type 1—successfully recruited young adults with T1D to a self-management education intervention [9]. However, these authors acknowledged that this approach introduces bias, since not all people with T1D choose to engage with these groups. Many analyses have concluded that digital recruitment introduces bias because internet browsing behavior correlates with demographics [13-15]. Therefore, any social media approach is inherently biased, but one potential strategy to diversify viewership is varying the way advertisements are delivered within the social media platform [10,13]. For example, advertisements can be placed within the home page news feed so they are viewed immediately or with unfocused scrolling, rather than having to intentionally visit a specific group page. Another strategy is *snowball sampling*, where initial respondents spread word to peers through social media and other web-based methods such as email [16]. Accordingly, evaluation of a multifaceted web-based recruitment campaign is important to determine the effectiveness of this approach for the T1D population.

### Objectives

Our overall objective was to describe recruitment engagement occurring within various web-based and in-person spaces to investigate the potential for selection bias and threats to external validity when recruiting adults with T1D. We addressed this objective via a substudy analyzing recruitment strategies for a parent study that was focused on a 10-week mobile exercise intervention for inadequately active adults with T1D. The intervention in the parent study used a customized mobile digital app—GlucoseZone (Fitscript LLC)—to provide on-demand instructional exercise videos, access to a text-based exercise coach with expertise in T1D, daily electronic self-monitoring diaries, and monthly data reports from a continuous glucose monitor (CGM) and an exercise smartwatch (Apple Watch 3) that were discussed with their coach in a motivational enhancement therapy session. The feasibility, acceptability, and efficacy of the intervention will be published in forthcoming manuscripts. The specific aims of this substudy were to (1) evaluate the effectiveness and cost of using news feed advertisements, snowball sampling, and an in-person approach at clinical visits to reach inadequately active adults with T1D for a mobile lifestyle intervention and (2) compare sociodemographic and clinical characteristics among responders against normative data.

## Methods

### Overview

The methods described below are based on our previous investigations of web-based advertising for different populations (ie, heavy-drinking smokers and heavy-drinking young adults with sleep concerns) [17,18]. Our previous studies and this study share the primary objective of evaluating the effectiveness and cost of using web-based advertising to recruit from the population of interest. This study compared social media news feed advertising, in-person approach at clinic visits, web-based snowball sampling, referral from prior studies, and ClinicalTrials.gov postings.

### Screening Process Overview

The recruitment campaign targeted individuals who met eligibility criteria for the parent intervention study: 18 to 65 years of age, have T1D or other absolute insulin deficiency diabetes, report inadequate exercise patterns (<3 days per week) [2], interest in participating in a mobile exercise intervention (ClinicalTrials.gov Identifier: NCT04204733), own a smartphone, own and are adherent to a CGM (consistently capture ≥70% of possible readings) [2], and read and speak English. The intervention also required an Apple Watch 3, which was provided to each participant by the research team for the duration of the study. Volunteers with a chronic disease or injury requiring exercise adjustments outside the scope of the mobile intervention were not eligible to participate. Each advertising strategy presented a brief description of the study with an invitation to inquire for more details. Those inquiring were provided a more detailed overview of study requirements and confidentiality policies. Those responsive to this more-detailed overview completed eligibility screening, and those eligible were invited to complete an intake visit at the closest of our two research sites—New Haven or Trumbull, Connecticut (n=9)—or by televideo, which was mandated for participants enrolled after the start of the COVID-19 pandemic (n=11). All participants completed an informed consent process before their intake. For televideo intakes, consenting was done on a separate televideo call the prior week so that intake supplies (blood pressure monitor, scale, etc) could be mailed. The study, screening, and consent process were approved by the Yale University Institutional Review Board.

### Advertising Strategies

Participants were recruited for the parent study over a 9-month period between November 12, 2019, and August 9, 2020, with a target enrollment of 20 participants.

#### Method #1: Social Media News Feed Advertisements

**Figure 1:**
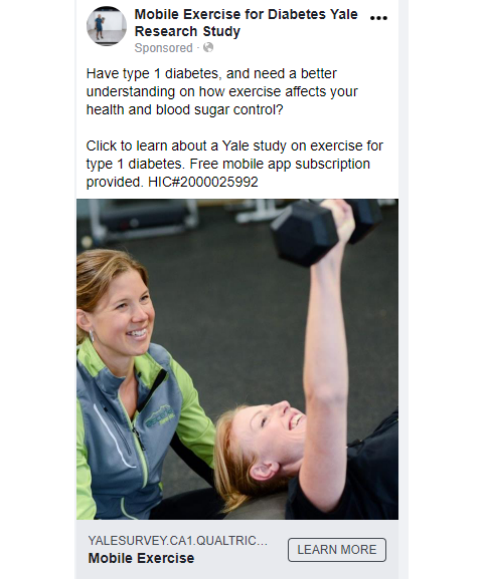
News feed advertisement to reach inadequately active adults with type 1 diabetes.

We ran an advertisement (Figure 1) through the paid news feed advertising platform of Facebook, which also includes Instagram, for 20 days total over five windows that were set according to times for which we had the capacity to enroll new volunteers (December 6-14, 2019; May 27-30, 2020; July 19-27, 2020; August 2 and 3, 2020; and August 9, 2020), until our target number of volunteers (N=20) had been enrolled. The advertisement appeared on the landing page of the desktop and mobile versions of Facebook and Instagram of individuals in the target age group (18 to 64 years) who listed at least one interest related to diabetes from a list we constructed by searching Facebook: Cure Type 1 Diabetes, Certified Diabetes Educator, American Diabetes Association, International Diabetes Federation, World Diabetes Day, Joslin Diabetes Center, Cure Diabetes, or Medtronic Diabetes. We specified a spending limit of US $25 per day.

We restricted the geographic target to a range that made travel to our research offices feasible without compromising the daily number of times the advertisement was displayed (ie, impressions). This area was a 25-mile radius of our city (New Haven, Connecticut) or the adjacent one (Hartford, Connecticut). Although we pilot-tested advertisements in other states when the mandated transition from in-person to televideo methods occurred, they yielded no volunteers (Multimedia Appendix 1), so this analysis is restricted to advertising days in Connecticut.

Wording style was taken from our previous successful social media campaigns [17,18]. Facebook and Instagram run on a shared platform. The platform allocates advertising space using an *auction* process based on the spending *bid* of the advertiser, relevance to the user (ie, web analytic estimated rate of the user acting upon the advertisement), and advertisement quality (ie, past user experience survey results) [19]. We used the platform’s bid-optimizing algorithms targeting the lowest cost per click. The platform’s auctioning and bid optimization include the Instagram space. The platform monitored the number of impressions, total reach (ie, number of people seeing the advertisements), advertisement clicks, and total cost for all advertisements. These data allowed us to evaluate efficacy and cost-effectiveness. 

By clicking the advertisement, inquirers were directed to a Health Insurance Portability and Accountability Act–compliant webpage (Qualtrics) that displayed an overview of the study: (1) a mobile app for people with T1D to manage exercise, which includes a free 3-month subscription to the mobile app, text-based coaching, a daily mobile diary, and feedback from automated devices (ie, Apple Watch, CGM, and insulin device); (2) 1-hour health assessments and surveys to be completed at the beginning and end of the 3 months; and (3) compensation for participation (US $100), research team contact details, and our confidentiality policy. Inquirers were informed that they could telephone the research team to obtain more information and complete eligibility screening to enroll, or leave their contact details and preferred times to be contacted in a secure webform on the website so that the research team could contact them. Those completing the webform received an email from the research team 1 business day later that confirmed receipt of their inquiry, were provided with a copy of the study overview from the website so that they could review it further as desired, and were notified that they would be telephoned 2 business days later so that they could ask questions about the study and complete eligibility screening. Those who answered or returned this telephone call were considered to be responsive volunteers.

#### Method #2: In-Person Approach at Clinical Visits

The Yale clinic serving adult patients with T1D did not permit in-person recruitment by researchers, and remote recruitment methods through clinic channels (ie, MyChart) were shut down at the time of our recruitment. Therefore, clinic recruitment was restricted to young adults attending the Yale Children’s Diabetes Clinic (ie, those 18 to 24 years old). The principal investigator (PI) (author GIA) successfully recruited a cohort of volunteers from this clinic for a prior study [20] and followed the same protocols for this study. Using medical record review, the PI identified candidates who met the age and T1D diagnosis criteria with appointments between November 12 and December 20, 2019 (ie, 27 days of clinic operation). The initial in-clinic approach occurred in the exam rooms over a 10-minute window before or between interactions with the diabetes provider, which were coordinated with the provider in advance, and utilized the following procedures. The PI knocked on the door. Once receiving the candidate’s permission to enter, the PI said, “Hello! I’m a researcher from Yale. We are doing a study on exercise for type 1 diabetes. It provides a free subscription to a mobile application for improving understanding on how exercise affects your health and blood sugar control. Would you like to have more information?” Candidates answering affirmatively were considered to be *inquirers*, analogous to clickers in method #1. The PI verbally reviewed a handout that mirrored method #1 regarding visuals (ie, Figure 1) and content (ie, the study overview webpage) and invited them to ask further questions and complete eligibility screening if they wanted to participate. Those electing to complete screening were considered to be responsive volunteers. Screening was completed immediately in person at the clinic setting. Clinic recruitment was discontinued after December 2019 due to its relative inefficiency (as described in the Results section) and COVID-19 pandemic restrictions.

#### Method #3: Web-Based Snowball Sampling

Two physically active individuals with T1D—both white non-Hispanic women in the 35-to-65-year age cohort—approached us volunteering to spread information about the study by word-of-mouth from April 9 through May 27, 2020, after learning about it through ClinicalTrials.gov or by word-of-mouth from members of our department. They targeted peer audiences, including a nationwide email list of personal friends with T1D, T1D support groups on Facebook (eg, Phoenix Valley T1D and Honest Exchange), and friends viewing their Facebook profile wall. They reported that they initially posted a link to the ClinicalTrials.gov page couched in a description that they personalized according to the venue (eg, a posting on a support group may have referenced a discussion at that group’s last meeting about the importance of exercise), which they followed up with personal exchanges with venue members as needed. These posts and exchanges occurred within private Facebook and email groups and were not monitored by the research team. Interested volunteers could inquire about the study by phone or email through study team contact details available through the role models or the ClinicalTrials.gov page. These *inquirers*, analogous to clickers in method #1, received the same series of responses from the research team as the webform completers: a study overview email 1 business day later and a telephone call 2 business days later, and those answering or returning this telephone call were considered to be responsive volunteers.

#### Other Methods

Over the course of the 9-month recruitment window, 3 participants in a prior Yale study for T1D [21] expressed interest in volunteering for further studies, and 4 viewers of the study on ClinicalTrials.gov emailed us requesting more information. These 7 people were considered to be *inquirers*, analogous to clickers in method #1. They received the same series of communications from the research team as the webform completers: a study overview email and a telephone call 2 business days later, and those answering or returning this telephone call were considered to be responsive volunteers.

### Eligibility Screening

Volunteers completed the eligibility interview with the PI (GIA, an exercise physiologist) by telephone or in person in the clinic setting depending on the mode of recruitment. The interview began with one question from the Paffenbarger Physical Activity Questionnaire that queries weekly frequency of regular activity sufficient to work up sweat, heart thumping, or out of breath [22]. Those responding 3 or more times per week were not eligible to participate.

The second part of the interview included a medical history based on the Physical Activity Readiness Questionnaire [23]. It captured all the volunteers’ chronic medical conditions, mobility limitations, medications, and other possible contraindications to exercise within the offerings of the mobile app (eg, chest pain and dizziness). All positive responses were reviewed by the study physician (author SAW) to rule out exclusion criteria.

### Cost-Effectiveness

Costs associated with each method are detailed in Table 1.

**Table 1:** Costs associated with each recruitment method used in the study.

Recruitment stage	Recruitment method tasks and their costs (US $)			
	News feed	Clinic	Snowball sampling	Other methods
Start-up	2 hours ($64.76^a^) to select image, slogan, and Facebook campaign settings $125.00 to have HIPAA^b^-compliant webform^c^	1 hour ($32.38) to design flyer	30 minutes ($16.19) to explain study to each of two snowball sample leaders ($32.38 total)	30 minutes ($16.19) to discuss referral system with principal investigator of previous study
Display advertisement	$0.012 per viewer for Facebook impression	1.14 minutes ($0.62) to screen chart^d^ 30 minutes ($16.19) per viewer to wait in clinic and find opportunity to approach participant	$0.00 (done by snowball sample leaders)	$0.00 (combined into below email that arranged screening)
Provide more information to inquirers^e^	$0.00 for initial clickers (directed automatically to webform page) 5 minutes ($2.70) per webform completer to send email template and follow up by phone^e^	1 color flyer ($0.20) per inquirer 5 minutes ($2.70) per inquirer to verbally explain study and answer questions	5 minutes ($2.70) per inquirer to send email template and follow up by phone^e^	5 minutes ($2.70) per inquirer to send email template and follow up by phone^e^
Screening session with responsive volunteers	15 minutes ($8.10) per volunteer to answer further questions about study and ask screening questions	15 minutes ($8.10) per volunteer to answer further questions about study and ask screening questions	15 minutes ($8.10) per volunteer to answer further questions about study and ask screening questions	15 minutes ($8.10) per volunteer to answer further questions about study and ask screening questions

^a^Personnel rate of $32.38/hour based on principal investigator’s salary + fringe.

^b^HIPAA: Health Insurance Portability and Accountability Act.

^c^Reflects 1 month of institutional subscription to Qualtrics (simulates larger trial where 20 days of advertising would occur in a single month).

^d^Calculated as 1 minute per chart / (88% of charts meeting age and type 1 diabetes diagnosis criteria) = 1.14 minutes per qualifying chart.

^e^Individuals not responding to first phone call were considered unresponsive.

### Participant Characteristics

The assessments below were taken from the intake visit and used for comparisons to normative data.

#### Baseline Exercise Levels

The physical activity question in the eligibility screening was followed at the intake appointment by the more granular timeline follow-back for exercise, in which volunteers were asked to recall exercise (ie, type, duration, and Borg Rating of Perceived Exertion scale [24]) for each calendar day going back 60 days using calendar prompts and memory aids (eg, holidays). This assessment has test-retest reliability (*r*=0.79-0.97) and convergent validity with weekly exercise logs (*r*=0.65-0.80) [25]. It was chosen since the parent study is a longitudinal design, thus benefitting from weekly repeated measures as opposed to other physical activity questionnaires that offer snapshots.

#### Demographics

Participants completed a REDCap (Research Electronic Data Capture) form (Vanderbilt University) at the intake appointment. It included age, gender, income, years of education, race, ethnicity, type and duration of diabetes, and mode of therapy (ie, continuous subcutaneous insulin infusion pump or multiple daily injections).

#### Glycemic Control

Hemoglobin A1c (HbA_1c_) was assessed by finger prick using the AccuBase A1c Home Test Kit (DTI Laboratories), a US Food and Drug Administration–approved method in which the user captures blood at home via capillary tube, injects the blood into EDTA preservative, and mails it to a central laboratory for analysis by high-performance liquid chromatography.

To save supply costs, participants who completed intake at a facility with a point-of-care HbA_1c_ machine available—DCA Vantage Analyzer (Bayer)—used it instead of the more expensive home test method. These 4 participants were 0.5 to 1.6 percentage points away from the classification cutoff used for analysis (7.0%), so differences between HbA_1c_ methods (typically ≤0.2 percentage points) did not impact results. Moreover, only 2 of these 4 participants ended up involved in the comparison between methods, and these participants were 0.9 to 1.6 percentage points away from the classification cutoff.

#### Resting Blood Pressure

Resting blood pressure was taken by averaging two brachial artery measurements from the seated position after at least 5 minutes of quiet rest using the Omron BP760N (Omron Healthcare), which includes a rigid cuff that minimizes fitting errors [26]. If the measurements differed by >5 mm Hg, then a third was taken and the closest two were averaged. On the day of the test, participants were asked to avoid confounders of blood pressure, including caffeine, exercise, alcohol, and tobacco, which was verbally confirmed before the test was taken. In accord with registry practices, we defined elevated blood pressure as ≥140/90 mm Hg regardless of medication treatment, since medication treatment can be a poor indicator of hypertension status in this population [27].

The PI manually applied the blood pressure cuff and supervised measurements at the in-person intakes (n=9), and instructed participants to assess themselves by live televideo for the remote intakes (n=11) [26]. All 4 participants with elevated blood pressure were among the latter group, meaning the results were not impacted by “white coat” hypertension (ie, elevation of blood pressure unique to the medical office setting). The less common “masked” hypertension phenomenon (ie, elevation of blood pressure unique to the nonoffice setting) could not be ruled out as a confounder.

#### Body Mass Index

Weight was taken in kilograms by the Body weight scale (Withings) in light clothing without shoes. Height was self-reported in feet and inches during phone screening and converted to meters. Body mass index was calculated, and values ≥30.0 kg/m^2^ were considered obese. Values within 3.0 kg/m^2^ of the obesity cutoff were confirmed at the intake visit using a seca 213 portable stadiometer.

### Normative Data

We obtained normative data to compare with our participants from the most recent (2016-2018) T1D Exchange Registry reports, a network of 70 US-based endocrinology practices that have enrolled 26,000 patients with T1D to complete a comprehensive questionnaire and grant access to their medical records [27-29].

### Statistical Analysis

Analyses were conducted using a significance level of α<.05. Data were tabulated in SPSS, version 26 (IBM Corp), and analyzed by the R software environment (The R Foundation).

#### Evaluation of Recruitment Effectiveness and Cost

For each recruitment method, we calculated the success proportion and cost at each conversion stage of recruitment: (1) viewers to inquirers (clickers of a news feed advertisement or people who contacted the research team in response to another form of advertisement), (2) inquirers to responsive volunteers (those who volunteer to participate after reviewing more information), and (3) responsive volunteers to eligible volunteers (those who pass screening) [13]. Proportions were compared between methods using chi-square tests (Fisher-Freeman-Halton if any cells <5), followed by post hoc pairwise comparisons using chi-square tests (Barnard test if any cells <5) with Benjamini and Hochberg false discovery rate–adjusted P values. We chose these tests over more conservative alternatives (ie, Fisher exact and Bonferroni-adjusted *P* values), since the small cell sizes presented a risk of type II error. *Other* methods were grouped for reporting and were not compared. Costs differed by magnitudes between methods—and some were nil—so were compared qualitatively [30]. Within each method, we compared demographic groups (ie, age and gender), since the study sought to increase age scope from previous reports to include adults 35 to 65 years old. Among the age brackets offered by Facebook analytics, *18 to 24 years* had just 5 clickers (0 enrollees) so was grouped with *25 to 34 years*. Pandemic status (ie, prepandemic vs midpandemic) was similarly tested as a possible confounder.

#### Comparison of Participant Characteristics to Normative Data

Demographic and clinical characteristics were compared using socially and clinically meaningful binary categories by testing whether the normative data proportion fell within the 95% CI of the proportion of our total sample and each recruitment method, excluding methods with ≤2 enrollees. Note that all of these enrolled cohorts were compared to the normative data but not each other.

## Results

### Evaluation of Recruitment Effectiveness and Cost

#### Method #1: Social Media News Feed Advertisements

The news feed advertisement was displayed 28,274 times (ie, impressions) for a total Facebook charge of US $328.85. Most of these impressions occurred on mobile devices (27,614/28,274, 97.67% vs 659/28,274, 2.33% on desktops). The advertisement was more successful on Facebook than on Instagram (US $1.19 vs US $1.46 per unique click), such that the bid-optimizing algorithm targeted most impressions (24,590/28,274, 86.97%) to the former. The number of unique viewers (n=11,738) was just 0.49% of the Facebook and Instagram users 18 to 64 years old in our geographic area (n=2,240,000), but 65.58% of those with at least one diabetes-related interest (n=17,900).

**Figure 2:**
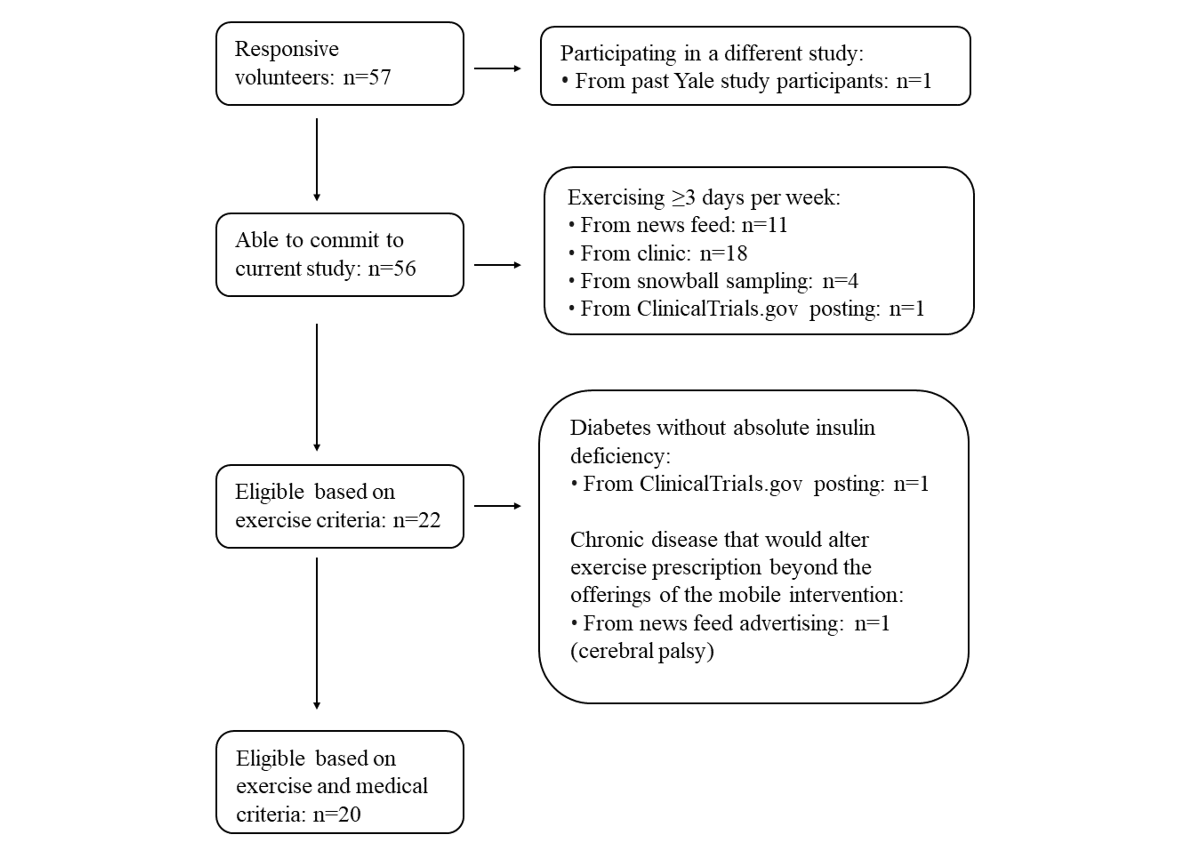
Participant screening flowchart.

Among the 11,738 viewers, 274 (2.33%) clicked the advertisement. Among them, 32 (11.7%) expressed some further interest by completing the webform (n=31 after removing 4 blanks or duplicates) or calling research staff (n=1). When research staff contacted these 32 people to provide more information, 11 (34%) did not return the contact and 1 (3%) stated that he could not make the time commitment to the study. The remaining 20 out of the 274 who clicked (7.3%) volunteered to participate, and 8 out of the 20 who volunteered (40%) were eligible (Figure 2).

Click rate was approximately 2× higher among women than men (Table 2). To ensure that this difference did not lead to unbalanced enrollment, we set male gender as an additional targeting filter on 4 out of 20 days, resulting in more impressions among men than women and, thus, a similar number of inquirers (ie, clickers) between the genders. Rates of volunteering and eligibility were not different by gender, so the final cohort of eligible volunteers was gender-balanced.

Age did not impact engagement success at any stage of the recruitment process, but the number of impressions (ie, the *overall denominator*) was approximately 7× higher among middle-aged than younger adults, as was the final number who were eligible. Facebook estimates that middle-aged adults outnumber younger adults within the subset of their users we targeted (ie, 13,700 vs 4400), and it is also possible they spend more time on the site. Among participants who clicked the advertisement, younger ones tended to complete the webform more often, but this tendency neither reached significance nor reflected any tendency to actually volunteer more often.

The pandemic period featured less expensive impressions (ie, fewer or less relevant competing advertisements) but also lower click rates, so the cost of enrolling a participant approximately doubled from the prepandemic period. The change in cost during the pandemic was similar for both genders (data not shown), and age could not be compared across time, since there was just 1 enrollee in the 18-to-34-year age category.

People clicking the advertisement on weekends tended to volunteer for the study approximately 2× more often than those who clicked during the week, but this difference neither reached significance nor impacted the final cost of enrolling a participant (US $95.88 on weekdays vs US $93.91 on weekends).

**Table 2:** News feed advertising details and costs by subgroups.

Recruitment metric		Gender^a^			Age (years)			Time relative to the pandemic			Day of the week	
		Men	Women	18-34		35-64	Before		During	Weekday		Weekend
**Facebook costs**												
	Estimated target audience, n	6300	11,700	4400		13,700	17,900		17,900	17,900		17,900
	Money spent, US $	192.22	134.95	28.60		306.84	131.34		197.51	191.43		143.36
	Impressions, n	18,655	9521	3264		23,872	8313		19,961	17,086		11,730
	Cost per impression, US $	0.010	0.014	0.009		0.013	0.016		0.010	0.011		0.012
	Unique viewers, n	6765	4924	1331		9652	3165		8573	N/A^b^		N/A
	Cost per unique viewer attracted, US $	0.028	0.027	0.021		0.032	0.041		0.023	N/A		N/A
	Clickers, n (% of unique viewers)	116 (1.7)	156 (3.2)^c^	32 (2.4)		242 (2.5)	116 (3.7)^c^		158 (1.8)	165^d^		113^d^
	Cost per click attracted, US $	1.66	0.97	0.89		1.34	1.13		1.25	1.16		1.27
	Completers of webform, n (% of clickers)^e^	14 (12.1)	18 (11.5)	7 (21.9)		25 (10.3)	11 (9.5)		21 (13.3)	15 (9.1)		17 (15.0)
	Responsive volunteers, n (% of clickers)^f^	9 (7.8)	11 (7.1)	3 (9.4)		17 (7.0)	10 (8.6)		10 (6.3)	7 (4.2)		11 (9.7)
	Cost per responsive volunteer attracted, US $	21.36	12.27	9.53		18.05	13.13		19.75	31.08		15.88
	Eligible volunteers, n (% of responsive volunteers)^g^	4 (44.4)	4 (36.4)	1 (33.3)		7 (41.2)	5 (50.0)		3 (30.0)	4 (57.1)		4 (36.4)
	Cost per eligible volunteer attracted, US $	48.06	33.74	28.60		43.83	26.27		65.84	47.86		35.84
**Other costs, US $**												
	Start-up^h^	94.88	94.88	94.88		94.88	94.88		94.88	94.88		94.88
	Contacting webform completers	37.80	48.60	18.90		67.50	29.70		56.70	40.50		45.90
	Screening responsive volunteers for eligibility	72.90	89.10	24.30		137.70	81.00		81.00	56.70		89.10
Total costs: cost per eligible volunteer enrolled, US $		99.45	91.88	166.68		86.70	67.38		143.36	95.88		93.31

^a^Excludes viewers with uncategorized gender (43/11,738, 0.4%); 2 out of these 43 viewers (4.7%) clicked the advertisement and 0 volunteered for the study.

^b^N/A: not applicable; this value was not traceable.

^c^Higher for women vs men (χ^2^_1_=25.9, *P*<.001) and before vs during pandemic (χ^2^_1_=32.9, *P*<.001), but not different by age (χ^2^_1_=0.02, *P*=.89).

^d^The percentage cannot be calculated because the number of unique viewers (ie, the denominator) was not traceable.

^e^Not different by any of the categories (gender: χ^2^_1_<0.001, *P*>.99; age: χ^2^_1_=2.6, *P*=.11; time: χ^2^_1_=0.6, *P*=.44; weekday vs weekend: χ^2^_1_=1.8, *P*=.18).

^f^Proportion of clickers volunteering (called *conversion* in the literature). It was not different by any of the categories (gender: χ^2^_1_<0.001, *P*>.99; age: Barnard test *P*=.89; time: Barnard test *P*=.60; weekday vs weekend: χ^2^_1_=2.5, *P*=.11).

^g^Not different by any of the categories (gender: Barnard test *P*=.79; age: Barnard test *P*=.91; time: Barnard test *P*=.43; weekday vs weekend: Barnard test *P*=.53).

^h^Start-up costs (Table 1) covered all participants, so were divided evenly between the two categories of each comparison.

#### Method #2: In-Person Approach at Clinic Visits

Among the 40 candidates who were approached, 32 (80%) were interested to hear about the study. After hearing the study overview, 12 of them declined to participate (4 due to the time commitment, 5 due to the CGM requirement, 1 due to the requirement to complete daily mobile diaries, and 2 provided no reason); the remaining 20 out of 32 inquirers (63%) volunteered to participate. Among them, 18 were excluded because they were already regularly exercising (Figure 2). The remaining 2 participants out of 20 volunteers (10%) were eligible and enrolled. Stratifying the results by gender revealed no differences in uptake at any stage (Table S1 in Multimedia Appendix 2), the age was uniformly 18 to 24 years old as stated in the Methods, and the time period for this strategy was exclusively prepandemic.

#### Method #3: Web-Based Snowball Sampling

Snowball sampling generated 13 volunteer inquiries by email, among whom 12 (92%) responded when the PI followed up by telephone. Among these, 4 were excluded because they were already regularly exercising. The remaining 8 out of 12 volunteers (67%) were eligible and enrolled. Stratifying the results by gender or age revealed no differences in uptake at any stage (Table S2 in Multimedia Appendix 2), and the only time this strategy was employed was midpandemic. These participants resided in seven different states, unlike the other recruitment methods, which restricted targeting to Connecticut.

#### Other Methods: Referral From Prior Study and ClinicalTrials.gov Posting

These methods yielded 7 inquiries, among whom 5 volunteered to participate (71%). Among them, 3 were disqualified (Figure 2). The other 2 out of 5 volunteers (40%) were eligible and enrolled.

#### Comparison Between Methods

As expected, the cost of a unique viewer was lower when approached by news feed advertisement versus in clinic (US $0.028 vs US $16.81) (Table 3). On the other hand, news feed advertisements were less likely than in-person clinic approaches to yield inquiries about the study (274/11,738, 2.33% vs 32/40, 80%; *P*<.001) or responsive volunteers from those inquiries (20/274, 7.3% vs 20/32, 63%; *P*<.001). However, responsive volunteers from news feed advertisements were more likely than those from in-person clinic approaches to be eligible for the study (8/20, 40% vs 2/20, 10%; *P*=.03). Thus, the overall cost of 1 eligible volunteer was approximately 5× lower when approached by news feed versus in clinic (US $95.88 vs US $479.79).

Snowball sampling was more likely than news feed and clinic methods to convert inquirers to responsive volunteers and responsive volunteers to eligible volunteers. Although the latter comparison was only significant against the clinic recruitment (8/12, 67% vs 2/20, 10%; *P*<.001), overall, these differences combined with its low start-up and personnel costs meant snowball sampling was 4× to 23× less expensive than news feed and clinic methods.

**Table 3:** Comparison of recruitment methods.

Recruitment metric			Recruitment method							
			News feed		Clinic		Snowball sampling		Other methods	
Days of action, n			20		27		48		271	
**Direct incremental marketing costs**										
	Money spent, US $	328.85		672.40		0.00		0.00		
	Impressions, n	28,274		40		N/A^a^		N/A		
	Cost per impression, US $	0.012		16.81		0.00		0.00		
	Unique viewers, n	11,738		40		N/A		N/A		
	Cost per unique viewer attracted, US $	0.028		16.81		0.00		0.00		
	Inquirers^b^, n (% of unique viewers)	274 (2.3)		32 (80.0)^c^		13^d^		7^d^		
	Cost per inquirer attracted, US $	1.20		21.01		0.00		0.00		
	Completers of webform, n (% of inquirers)	32 (11.7)		N/A		N/A		N/A		
	Responsive volunteers^e^, n (% of inquirers)	20 (7.3)		20 (62.5)^c^		12 (92.3)^f,g^		5 (71.4)		
	Cost per responsive volunteer attracted, US $	16.44		33.62		0.00		0.00		
	Eligible volunteers^h^, n (% of responsive volunteers)	8 (40.0)		2 (10.0)^i^		8 (66.7)^g^		2 (40.0)		
	Cost per eligible volunteer attracted, US $	41.11		336.20		0.00		0.00		
**Other costs, US $**										
	Start-up	189.76		32.38		32.38		16.19		
	Contacting and explaining study to inquirers	86.40		92.80		35.10		18.90		
	Screening responsive volunteers for eligibility	162.00		162.00		97.20		40.50		
Total costs: cost per eligible volunteer enrolled, US $			95.88		479.79		20.59		34.92	

^a^N/A: not applicable; this value was not traceable.

^b^Defined as person who clicks (news feed advertisement) or requests more information from the research team (other recruitment methods).

^c^Greater than news feed by chi-square (inquirers: χ^2^_1_=919.8, *P*<.001; responsive volunteers: χ^2^_1_=72.1, *P*<.001).

^d^The percentage cannot be calculated because the number of unique viewers (ie, the denominator) was not traceable.

^e^Refers to proportion of clickers volunteering (called *conversion* in the literature) (3-way *P*<.001).

^f^Greater than news feed by Barnard test (*P*<.001).

^g^Greater than clinic by Barnard test (response rate: *P*=.048; eligibility: *P*<.001).

^h^3-way *P*=.003. Not significant for news feed versus snowball sampling (Barnard test *P*=.23).

^i^Less than news feed by Barnard test (*P*=.03).

### Comparison of Participant Characteristics to Normative Data

The sample characteristics are given in Table 4. Most enrolled participants (18/20, 90%) had T1D, and the rest (2/20, 10%) had latent autoimmune diabetes of adulthood. The sample was gender-balanced with an average age of 42.3 (SD 15.0) years. Most participants were Caucasian (19/20, 95%), had completed a 4-year college degree (14/20, 70%), and had a household income greater than US $50,000 per year (17/20, 85%). The majority of participants (17/20, 85%) managed their diabetes with a continuous subcutaneous insulin infusion pump, with 3/20 (15%) using multiple daily injections. All used a CGM in accordance with inclusion criteria. Over half of the participants (12/20, 60%) had HbA_1c_ above target (ie, HbA_1c_ ≥7.0%). Half (10/20) were exercising an average of less than 0.5 days per week, and half (10/20) had obesity. A smaller fraction had uncontrolled blood pressure (20%). In comparison to the T1D Exchange Registry, the sample overrepresented low exercise, HbA_1c_ meeting target, and obesity. Division by recruitment methods revealed that news feed advertising overrepresented obesity and older age, whereas snowball sampling overrepresented HbA_1c_ meeting target and low exercise.

**Table 4:** Sample characteristics of the full enrolled cohort and subsets for each method, each compared against normative data from the T1D Exchange Registry.^a^

Characteristic	Full enrolled cohort (N=20)			Subset enrolled from news feed (n=8)			Subset enrolled from snowball sampling (n=8)		Normative data from T1D Exchange Registry, n (%)
	n (%)	95% CI of %	n (%)		95% CI of %	n (%)		95% CI of %	
Age (≥50 years)	9 (45)	23-68	6 (75)		35-97^b^	3 (38)		9-76	3445/11,919 (29)
Sex (female)	11 (55)	32-77	5 (63)		24-91	4 (50)		16-84	6188/11,919 (52)
Race or ethnicity (Caucasian)	19 (95)	75-100	8 (100)		63-100	8 (100)		63-100	10,134/11,841 (86)
Education (bachelor’s degree or higher)	14 (70)	46-88	6 (75)		35-97	6 (75)		35-97	5669/11,054 (51)
Advantaged income (≥US $50,000)	17 (85)	62-97	6 (75)		35-97	8 (100)		63-100	6112/8575 (71)
Pump therapy	17 (85)	62-97	7 (88)		47-100	7 (88)		47-100	7371/11,785 (63)
Continuous glucose monitor use	20 (100)	83-100^b^	8 (100)		63-100^b^	8 (100)		63-100^b^	1685/6564 (26)
Duration of diabetes (<10 years)	7 (35)	15-59	2 (25)		3-65	3 (38)		9-76	2436/11,901 (20)
Hemoglobin A_1c_ (≥7.0%)	12 (60)	36-81^b^	7 (88)		47-100	2 (25)		3-65^b^	4851/6181 (78)
Low exercise (<0.5 days/week)	10 (50)	27-73^b^	3 (38)		9-76	5 (63)		24-91^b^	848/7153 (12)^c^
Obesity (BMI ≥30.0 kg/m^2^)	10 (50)	27-73^b^	7 (88)		47-100^b^	2 (25)		3-65	2571/10,204 (25)
Uncontrolled blood pressure	4 (20)	6-44	1 (13)		0-53	3 (38)		9-76	1648/11,697 (14)

^a^The enrolled cohort and each subset were compared against normative data but not each other.

^b^The 95% CI of the study cohort or subset does not include normative value, indicating bias.

^c^Taken from 2010-2012 iteration of the T1D Exchange Registry, since not yet published for 2016-2018 iteration.

## Discussion

### Principal Findings

This substudy evaluated the effectiveness, cost, and demographic representation achieved by web-based and in-person recruitment strategies for enrolling inadequately active adults aged 18 to 65 years with T1D into a mobile exercise intervention. The strategies collectively achieved cost-effective recruitment of adults that met our inclusion criteria of CGM users with inadequate baseline exercise patterns. Snowball sampling was the most cost-effective method and reached participants with exceptionally low exercise levels, but it overrepresented individuals with optimal glycemic control. We also tested other methods, including social media news feed advertising and in-person clinic recruitment. Among these methods, news feed advertising was more cost-effective than clinic recruitment, with a yield rate that would be satisfactory for a large clinical trial (1 participant per 2 to 3 days of advertising). Its initial engagement of men was more challenging than of women, but this was easily addressed by directing more impressions to men, since their responsiveness and eligibility were equal to women once they clicked the advertisement. Although prior literature found that social media is less effective for recruiting middle-aged and older adults compared to young adults [13], we observed that it was easier to target the middle-aged and older population because a greater number of them had diabetes-related profile interests. These results justify the previously highlighted need to diversify recruitment strategies [13-15] by including online methods and a variety of advertisement delivery modes within those methods.

The underrepresentation of elevated average blood glucose (ie, above-target HbA_1c_) by snowball sampling led to a similar bias in the final cohort, which is problematic since such individuals have increased risk of mortality due to cardiovascular disease, and exercise can make blood glucose go too high [2] without proper guidance by an exercise intervention such as ours. Another contributor to this bias may have been the inclusion criteria of owning a CGM, which is associated with better glycemic control [2]. The final cohort also overrepresented low exercise levels and obesity, but these differences are inherent to the research question, since inadequate exercise was an inclusion criterion and leads to risk of obesity in the T1D population [29,31].

### Comparison With Previous Work

Online forums have many uses in the T1D community, including emotional support [11], promotion of events, circulation of educational resources [32], and interactive technical support from peers and mentors with diabetes technology [11]. Snowball sampling or direct messages on media produced by these forums were, therefore, low cost and high return, although they were demographically biased recruitment strategies in our study (ie, overrepresenting optimal glycemic control and possibly other factors beyond our statistical power) and in previous work (ie, overrepresenting women and college education) [9,11,33,34]. Others have used news feed advertising for young adults [10], a strategy we successfully extended to middle-aged and older adults but failed to reproduce among the younger adults.

Young adulthood (ie, 18 to 34 years old) is a time of critical health and psychosocial concerns in T1D (eg, pregnancy, transition from pediatric to adult care, and parental to personal health insurance), but consensus statements recognize that this age group is understudied in clinical trials [2]. Successful strategies for reaching this group include targeting by the age listed on social media profile [10] or medical record [10,21], or online support groups specific to young adults [9]. We, unfortunately, did not design our web-based methods to achieve such targeting; our news feed advertisements were targeted based on diabetes-related profile interests, which were uncommon among young adults, and the individuals who volunteered to start our snowball sampling happened to be middle-aged rather than young adults. Nonetheless, the limited number of young adults who were reached by our advertisements—32 clicked on news feed advertisements and 3 inquired from snowball sampling—were equally, if not more, likely than their older counterparts to be responsive and eligible. Taken together with the relative inefficiency of recruiting young adults through our clinic, these data indicate that web-based recruitment is an important strategy for reaching young adults with T1D but requires careful targeting to ensure they are reached.

Compared with this limited literature on web-based recruitment for T1D interventions, clinic-based recruitment strategies are more common [10,35,36] and some have found that they are more effective than web-based recruitment [10]. We, however, found the opposite. Some contributing factors may not be generalizable to all other studies. First, we had remote data collection, whereas studies holding intervention sessions or laboratory tests at clinics may benefit from recruiting in the same clinic to target individuals accustomed to visiting it [10,35,36]. Second, our major exclusion criterion (ie, regular exercise at baseline) could not be screened on medical records, leading to high ineligibility rates. Third, we had restrictions on approaching candidates over 25 years old through clinic channels. Fourth, our clinic did not allow mailing lists, which had higher eligibility and cost-effectiveness than in-person clinic recruitment in previous studies of T1D and type 2 diabetes [10,37]. Even those authors, however, noted that the reach of a clinic-based mailing list is limited [10] compared to the large pool that social media can access quickly (eg, 11,738 viewers over 20 days in our study). Overall, our findings highlight that web-based recruitment for T1D warrants more exploration relative to the clinic-based channels, especially when clinic visits are not required for data collection.

News feed advertising on Facebook has demonstrated cost-effectiveness in previous research. In a systematic review of 35 studies that assessed cost, the median cost of enrolling an eligible candidate was US $14.41 [13], which is substantially less expensive than our result (US $95.88). There are several factors that likely contributed to this cost discrepancy, but the most substantial is likely that only 10 studies in the systematic review were clinical trials. In a review restricted to clinical trials, 6 out of 16 (38%) of the reported studies yielded a result more expensive than ours [38]. It is also noteworthy that we included costs outside of direct Facebook charges (eg, personnel time), which most studies reviewed did not [38].

The first review [13] also assessed other factors that can elevate costs: engagement (ie, clicks per impression), conversion (ie, responsive volunteers per click), and eligibility (ie, volunteers eligible per volunteers responsive). Our rates of engagement and eligibility were lower than those of prior studies, but our rate of conversion outscored most of the studies reviewed. In summary, the driver of our cost was the low rate of initial engagement (ie, click rate) and the low proportion of responsive volunteers who met the eligibility criteria. The low click rates may reflect the low proportion of the population affected by T1D (0.5%) [1]. We targeted broader diabetes-related interests, but it is likely many of the individuals did not have diabetes despite their interest, or had the more common type 2 diabetes. They would not have found the intervention study appealing. We also note that click rates became lower during the COVID-19 pandemic, which coincided with the onset of warmer weather. Therefore, our intervention may have been more appealing during colder weather and/or times when volunteers were following a more typical daily schedule without quarantine modifications. Unfortunately, we could not survey nonclickers for factors influencing their decision not to click. One speculative explanation is that warmer weather and school quarantines prompted adults to initiate outdoor activities with young relatives, thus, not needing a mobile intervention to guide their exercise.

The low eligibility, meanwhile, was caused by the exclusion criteria of exercising 3 or more days per week. This challenge is not surprising, since 33% of adults with T1D report exercising 5 or more days per week, and another 55% report exercising 1 to 4 days per week [29]. Other important factors may have accounted for the cost-effectiveness of the results. For instance, our study required participants to have a CGM and to participate in a 10-week intervention with a mobile phone. In comparison, only 26% of adults, nationally, currently use a CGM [28], and most previous studies required less volunteer commitment; most studies involved brief web-based assessments or interventions [13]. These factors could have attenuated engagement, conversion, and enrollment of our recruitment process and could have driven up costs.

A prior study [10] faced similar challenges of engaging adult viewers in an advertisement calling for those with diabetes—predominantly T1D, as they were young adults—and then screening for those who met additional criteria, in their case, suboptimal glycemic control (HbA_1c_ ≥8.0%) and low socioeconomic status. They achieved higher engagement than our study (ie, cost per click was US $0.45), but their conversion rate was lower (59/7031, 0.84%) and their eligibility rate was similar (27/59, 46%), such that the cost of enrolling one participant was three times higher (US $334). The engagement difference may be attributable to two differences in the targeting strategies. First, our study targeted advertisements to diabetes based on profile interests, whereas the prior study used *likes* of diabetes-related posts. Further study is required regarding the differing implications of these two virtual behavior characteristics; we can speculate that individuals *liking* posts are more inclined to actively engage with content (eg, by clicking) rather than passively viewing. Second, our study’s advertising theme was “understanding how exercise affects blood sugar control,” whereas the prior study’s themes were diabetes-related imagery, compensation, urgency and time running out, altruism, the study team’s empathy, call to action, and difficult aspects of managing diabetes. The eligibility rate similarity was expected, since this study and the previous study had criteria that applied to a minority of the T1D population: inadequate exercise [29] and low socioeconomic status [28], respectively. The conversion rate difference is more difficult to interpret, since the prior study did not report the contents of the landing page reached from clicking. However, the landing page is likely to include a description of the required assessments, compensation, and intervention offerings. Study requirements (ie, two visits with clinical and psychosocial assessments) and compensation (US $100 vs US $75) were similar, and all of our participants stated that compensation did not influence their desire to participate. Intervention offerings included a customized mobile digital app offering exercise coaching, biosensor feedback, and daily diary self-monitoring in this study versus occupational therapy for diabetes management in the prior study. In summary, although it was relatively challenging for us to initially attract clickers, the conversion to responsive volunteers of 7.3% was high compared to other studies of people with and without T1D, implying that mobile exercise support is appealing to people with T1D, and efforts to scale up its dissemination are warranted.

### Limitations

Limitations of this study should also be noted. First, we did not perform the complex social network mining required to trace the snowball sampling as carefully as we traced the news feed advertisements and clinic recruitment. Doing so might have lent insights into better targeting the snowball sampling, but would likely be resource intensive compared to the user-friendly tracking tools of the Facebook advertising dashboard. Second, the sample was too underpowered to address the representativeness of the enrolled cohorts. The data suggest that snowball sampling should be used cautiously because of the possibility to overrepresent optimal HbA_1c_, but there may be other differences between methods that were undetectable due to limited sample size, number of assessments, and stages of the recruitment process where they were taken. Third, the small sample left insufficient room to rotate strategies, such as the gender and activities of advertisement models and snowball sample leaders. In particular, we only featured women, whereas previous reports suggest that men are more effective at recruiting both genders [36]; also, weekend advertisement clicks tended to convert to responsive volunteers more frequently than weekday clicks, but this trend did not reach statistical significance as it might have with a larger sample. Fourth, the design was observational so cannot infer direction of associations. Fifth, a CGM was required for participation and we were only able to recruit those with a current CGM. Although CGM use increased in this population, nationally, from 7% in 2010-2012 to 26% in 2016-2018 [28], and is being urgently recommended by the standard of care [2], CGMs are still not used by the majority. The study also tended to overrepresent those using insulin pumps as opposed to multiple daily injections, which was perhaps related to the CGM requirement biasing toward people with greater technology uptake.

### Conclusions

Despite these limitations, this study demonstrated that web-based recruiting strategies targeting physically inactive adults with T1D are cost-effective and efficient compared to traditional methods, as well as similar strategies in other populations [38]. Adults with T1D are a hard-to-reach group and face several barriers (eg, fear of hypoglycemia, actual hypoglycemia, neuropathy, and social stigma) to achieving the target exercise recommendations of exercising at least every other day [2,39,40]. Thus, having another avenue for recruitment and anonymity (ie, the comfort of one’s own home) to participate in physical activity is essential. Data from this study lend insight into the scalability of this approach by demonstrating that web-based recruitment strategies are viable and steady channels for recruitment of individuals with T1D and other risk factors. Future studies should attempt tailoring of these methods to better reach vulnerable subgroups among people with T1D, including young adults, those with suboptimal glycemic control, and racial and economic minorities. Possible tailoring strategies could include snowball sampling starting with purposefully recruited individuals from these subgroups or news feed advertising through social media platforms besides Facebook (eg, Reddit and YouTube).

## Appendix

Multimedia Appendix 1 Results of advertisements outside Connecticut.

Multimedia Appendix 2 Stratification of clinic and snowball sampling results by demographics.

## Abbreviations

CGM: continuous glucose monitor
HbA_1c_: hemoglobin A_1c_
PI: principal investigator
REDCap: Research Electronic Data Capture
T1D: type 1 diabetes
VA: Veterans Affairs

## References

[1] Centers for Disease Control and Prevention (2020). National Diabetes Statistics Report, 2020: Estimates of Diabetes and Its Burden in the United States.

[2] American Diabetes Association (2021). 5. Facilitating behavior change and well-being to improve health outcomes: Standards of Medical Care in Diabetes—2021. Diabetes Care.

[3] Sporrel K, Nibbeling N, Wang S, Ettema D, Simons M (2021). Unraveling mobile health exercise interventions for adults: Scoping review on the implementations and designs of persuasive strategies. JMIR Mhealth Uhealth.

[4] Wadden TA, Tronieri JS, Butryn ML (2020). Lifestyle modification approaches for the treatment of obesity in adults. Am Psychol.

[5] Thomas JG, Goldstein CM, Bond DS, Lillis J, Hekler EB, Emerson JA, Espel-Huynh HM, Goldstein SP, Dunsiger SI, Evans EW, Butryn ML, Huang J, Wing RR (2021). Evaluation of intervention components to maximize outcomes of behavioral obesity treatment delivered online: A factorial experiment following the multiphase optimization strategy framework. Contemp Clin Trials.

[6] Bray GA, Heisel WE, Afshin A, Jensen MD, Dietz WH, Long M, Kushner RF, Daniels SR, Wadden TA, Tsai AG, Hu FB, Jakicic JM, Ryan DH, Wolfe BM, Inge TH (2018). The science of obesity management: An endocrine society scientific statement. Endocr Rev.

[7] Shubrook JH, Brannan GD, Wapner A, Klein G, Schwartz FL (2018). Time needed for diabetes self-care: Nationwide survey of certified diabetes educators. Diabetes Spectr.

[8] Loree JM, Anand S, Dasari A, Unger JM, Gothwal A, Ellis LM, Varadhachary G, Kopetz S, Overman MJ, Raghav K (2019). Disparity of race reporting and representation in clinical trials leading to cancer drug approvals from 2008 to 2018. JAMA Oncol.

[9] Wisk LE, Nelson EB, Magane KM, Weitzman ER (2019). Clinical trial recruitment and retention of college students with type 1 diabetes via social media: An implementation case study. J Diabetes Sci Technol.

[10] Salvy S, Carandang K, Vigen CL, Concha-Chavez A, Sequeira PA, Blanchard J, Diaz J, Raymond J, Pyatak EA (2020). Effectiveness of social media (Facebook), targeted mailing, and in-person solicitation for the recruitment of young adult in a diabetes self-management clinical trial. Clin Trials.

[11] White K, Gebremariam A, Lewis D, Nordgren W, Wedding J, Pasek J, Garrity A, Hirschfeld E, Lee JM (2018). Motivations for participation in an online social media community for diabetes. J Diabetes Sci Technol.

[12] Crocket H (2020). Peer mentoring in the do-it-yourself artificial pancreas system community. J Diabetes Sci Technol.

[13] Whitaker C, Stevelink S, Fear N (2017). The use of Facebook in recruiting participants for health research purposes: A systematic review. J Med Internet Res.

[14] Benedict C, Hahn AL, Diefenbach MA, Ford JS (2019). Recruitment via social media: Advantages and potential biases. Digit Health.

[15] Keaver L, McGough A, Du M, Chang W, Chomitz V, Allen JD, Attai DJ, Gualtieri L, Zhang FF (2019). Potential of using Twitter to recruit cancer survivors and their willingness to participate in nutrition research and web-based interventions: A cross-sectional study. JMIR Cancer.

[16] Johnston LG, Sabin K (2010). Sampling hard-to-reach populations with respondent driven sampling. Method Innov.

[17] Ash GI, Robledo DS, Ishii M, Pittman B, DeMartini KS, O'Malley SS, Redeker NS, Fucito LM (2020). Using web-based social media to recruit heavy-drinking young adults for sleep intervention: Prospective observational study. J Med Internet Res.

[18] Bold KW, Hanrahan TH, O'Malley SS, Fucito LM (2016). Exploring the utility of web-based social media advertising to recruit adult heavy-drinking smokers for treatment. J Med Internet Res.

[19] About ad auctions. Facebook for Business.

[20] Ash GI, Joiner KL, Savoye M, Baker JS, Gerosa J, Kleck E, Patel NS, Sadler LS, Stults-Kolehmainen M, Weinzimer SA, Grey M (2019). Feasibility and safety of a group physical activity program for youth with type 1 diabetes. Pediatr Diabetes.

[21] Griggs S, Redeker NS, Crawford SL, Grey M (2020). Sleep, self-management, neurocognitive function, and glycemia in emerging adults with type 1 diabetes mellitus: A research protocol. Res Nurs Health.

[22] Tonstad S, Herring P, Lee J, Johnson JD (2018). Two physical activity measures: Paffenbarger Physical Activity Questionnaire versus Aerobics Center Longitudinal Study as predictors of adult-onset type 2 diabetes in a follow-up study. Am J Health Promot.

[23] Warburton DER, Gledhill N, Jamnik VK, Bredin SSD, McKenzie DC, Stone J, Charlesworth S, Shephard RJ (2011). Evidence-based risk assessment and recommendations for physical activity clearance: Consensus document 2011. Appl Physiol Nutr Metab.

[24] Haddad M, Stylianides G, Djaoui L, Dellal A, Chamari K (2017). Session-RPE method for training load monitoring: Validity, ecological usefulness, and influencing factors. Front Neurosci.

[25] Panza GA, Weinstock J, Ash GI, Pescatello LS (2012). Psychometric evaluation of the timeline followback for exercise among college students. Psychol Sport Exerc.

[26] Whelton PK, Carey RM, Aronow WS, Casey DE, Collins KJ, Dennison Himmelfarb C, DePalma SM, Gidding S, Jamerson KA, Jones DW, MacLaughlin EJ, Muntner P, Ovbiagele B, Smith SC, Spencer CC, Stafford RS, Taler SJ, Thomas RJ, Williams KA, Williamson JD, Wright JT (2018). 2017 ACC/AHA/AAPA/ABC/ACPM/AGS/APhA/ASH/ASPC/NMA/PCNA Guideline for the Prevention, Detection, Evaluation, and Management of High Blood Pressure in Adults: Executive summary: A report of the American College of Cardiology/American Heart Association Task Force on Clinical Practice Guidelines. Hypertension.

[27] Shah VN, Grimsmann JM, Foster NC, Dost A, Miller KM, Pavel M, Weinstock RS, Karges W, Maahs DM, Holl RW (2020). Undertreatment of cardiovascular risk factors in the type 1 diabetes exchange clinic network (United States) and the prospective diabetes follow-up (Germany/Austria) registries. Diabetes Obes Metab.

[28] Foster NC, Beck RW, Miller KM, Clements MA, Rickels MR, DiMeglio LA, Maahs DM, Tamborlane WV, Bergenstal R, Smith E, Olson BA, Garg SK (2019). State of type 1 diabetes management and outcomes from the T1D exchange in 2016-2018. Diabetes Technol Ther.

[29] McCarthy MM, Whittemore R, Grey M (2016). Physical activity in adults with type 1 diabetes. Diabetes Educ.

[30] Zhou XH, Melfi CA, Hui SL (1997). Methods for comparison of cost data. Ann Intern Med.

[31] Brazeau AS, Leroux C, Mircescu H, Rabasa-Lhoret R (2012). Physical activity level and body composition among adults with type 1 diabetes. Diabet Med.

[32] Gabarron E, Larbi D, Dorronzoro E, Hasvold PE, Wynn R, Årsand E (2020). Factors engaging users of diabetes social media channels on Facebook, Twitter, and Instagram: Observational study. J Med Internet Res.

[33] Oser SM, Stuckey HL, Parascando JA, McGinley EL, Berg A, Oser TK (2019). Glycated hemoglobin differences among blog-reading adults with type 1 diabetes compared with those who do not read blogs: Cross-sectional study. JMIR Diabetes.

[34] Brazeau A, Nakhla M, Wright M, Henderson M, Panagiotopoulos C, Pacaud D, Kearns P, Rahme E, Da Costa D, Dasgupta K (2018). Stigma and its association with glycemic control and hypoglycemia in adolescents and young adults with type 1 diabetes: Cross-sectional study. J Med Internet Res.

[35] Amsberg S, Wijk I, Livheim F, Toft E, Johansson U, Anderbro T (2018). Acceptance and commitment therapy (ACT) for adult type 1 diabetes management: Study protocol for a randomised controlled trial. BMJ Open.

[36] Riddell M, Li Z, Beck RW, Gal R, Jacobs PG, Castle JR, Gillingham M, Clements MA, Patton SR, Dassau E, Doyle Iii FJ, Martin C, Calhoun P, Rickels M (2021). More time in glucose range during exercise days than sedentary days in adults living with type 1 diabetes. Diabetes Technol Ther.

[37] Johnson EJ, Niles BL, Mori DL (2015). Targeted recruitment of adults with type 2 diabetes for a physical activity intervention. Diabetes Spectr.

[38] Darmawan I, Bakker C, Brockman TA, Patten CA, Eder M (2020). The role of social media in enhancing clinical trial recruitment: Scoping review. J Med Internet Res.

[39] Kennedy A, Narendran P, Andrews RC, Daley A, Greenfield SM, EXTOD Group (2018). Attitudes and barriers to exercise in adults with a recent diagnosis of type 1 diabetes: A qualitative study of participants in the Exercise for Type 1 Diabetes (EXTOD) study. BMJ Open.

[40] Brazeau A, Rabasa-Lhoret R, Strychar I, Mircescu H (2008). Barriers to physical activity among patients with type 1 diabetes. Diabetes Care.

