# Supplementary material for: Evaluation of Web-Based and In-Person Methods to Recruit Adults With Type 1 Diabetes for a Mobile Exercise Intervention: Prospective Observational Study
Source: JMIR Diabetes. 2021 Jul 8;6(3):e28309. doi: 10.2196/28309 (PMC8299346; doi:10.2196/28309)
Supplement: Multimedia Appendix 2 [file diabetes_v6i3e28309_app2.docx]

| **Table S1.** Clinic recruitment by gender. | | |
| --- | --- | --- |
|  | **Men** | **Women** |
| Direct incremental marketing costs |  |  |
| Spent on impressions | $353.01 | $319.39 |
| Impressions | 21 | 19 |
| Cost per impression | $16.81 | $16.81 |
| Unique viewers | 21 | 19 |
| Cost per unique viewer attracted | $16.81 | $16.81 |
| Inquirers | 19 (90.5%) | 13 (68.4%) |
| Cost per inquirer attracted | $18.58 | $24.57 |
| Responsive volunteers | 13 (68.4%) | 7 (53.8%) |
| Cost per responsive volunteer attracted | $27.15 | $45.63 |
| Eligible Volunteers | 1 (7.7%) | 1 (14.3%) |
| Cost per eligible volunteer attracted | $353.01 | $319.39 |
| Other Costs |  |  |
| Startup | $16.19 | $16.19 |
| Contacting and explaining study to inquirers | $55.10 | $37.70 |
| Screening responsive volunteers for eligibility | $105.30 | $56.70 |
| Total Costs |  |  |
| Cost per eligible volunteer enrolled | $529.60 | $429.98 |
| No proportions were different by gender (inquirers Barnard’s *P* = .10, responsive volunteers χ^2^ (1) = 0.2, *P* = .64, eligible volunteers Barnard’s *P* = .93). | | |

| **Table S2.** Snowball sampling by gender and age. | | | | |
| --- | --- | --- | --- | --- |
|  | **Gender** | | **Age** | |
|  | **Men** | **Women** | **18-34 years old** | **35-64 years old** |
| Direct incremental marketing costs | $0.00 | $0.00 | $0.00 | $0.00 |
| Inquirers | 8 | 5 | 3 | 10 |
| Responsive Volunteers | 7 (87.5%) | 5 (100.0%) | 3 (100.0%) | 9 (90.0%) |
| Eligible Volunteers | 4 (57.1%) | 4 (80.0%) | 2 (66.7%) | 6 (66.7%) |
| Other costs |  |  |  |  |
| Startup | $16.19 | $16.19 | $16.19 | $16.19 |
| Contacting and explaining study to inquirers | $21.60 | $13.50 | $8.10 | $27.00 |
| Screening responsive volunteers for eligibility | $56.70 | $40.50 | $24.30 | $72.90 |
| Total costs |  |  |  |  |
| Cost per eligible volunteer | $23.62 | $17.55 | $24.30 | $19.35 |
| No proportions were different by gender (Barnard’s *P* responsive volunteers .81, eligible volunteers 55) or age (Barnard’s *P* responsive volunteers .98, eligible volunteers 1.00) | | | | |
